# A rare combination of anterior cerebral arterial ring with accessory middle and duplicated posterior cerebral arteries

**DOI:** 10.1007/s00276-026-03823-z

**Published:** 2026-02-13

**Authors:** George Triantafyllou, Savvas Melissanidis, Nikolaos-Achilleas Arkoudis, Panagiotis Papadopoulos-Manolarakis, George Tsakotos, Maria Piagkou

**Affiliations:** 1 Department of Anatomy, Faculty of Health Sciences, School of Medicine, National and Kapodistrian University of Athens, 75 Mikras Asias str, 11527 Goudi, Athens, Greece; 2 Radiological Clinic, Asklipios Medica, Veroia, Greece; 3 Research Unit of Radiology and Medical Imaging, National and Kapodistrian University of Athens, Athens, Greece; 4 Second Department of Radiology, General University Hospital “Attikon”, National and Kapodistrian University of Athens, Athens , Greece; 5 Department of Neurosurgery, General Hospital of Nikaia-Piraeus, Nikaia, Greece

**Keywords:** Cerebral arterial circle, Variation, Neuroradiology, Anatomy

## Abstract

**Purposes:**

To report a unique configuration of the cerebral arterial circle identified incidentally during magnetic resonance angiography (MRA).

**Methods:**

An 87-year-old female patient was evaluated using MRA on a 3-Tesla scanner with the time-of-flight (TOF) technique.

**Results:**

On the anterior circulation, an arterial ring was identified at the junction of the left anterior cerebral and anterior communicating arteries, suggesting a double origin of the left A2 segment. An accessory middle cerebral artery originated from the proximal left A1 segment. Additionally, the right A1 segment was aplastic, with the right A2 segment supplied by the left anterior cerebral. In the posterior circulation, the left posterior cerebral artery exhibited a fetal-type origin with a hypoplastic P1 segment. On the right side, a duplicated posterior cerebral artery originated from the internal carotid artery and supplied the temporal branch.

**Conclusions:**

This case presents a rare combination of an A2 double origin, accessory MCA, and PCA duplication, highlighting the morphological complexity of the cerebral arterial circle. Detailed preoperative assessment using volume-rendered MRA is essential to identify such complex variants and minimize intraoperative complications.

**Supplementary Information:**

The online version contains supplementary material available at 10.1007/s00276-026-03823-z.

## Introduction

The cerebral arterial circle is formed by the terminal branches of the internal carotid arteries (ICA) and the basilar artery (BA), along with their anastomoses.

Typically, each ICA bifurcates into the anterior and middle cerebral arteries (ACA and MCA), and the BA bifurcates into the two posterior cerebral arteries (PCA). The bilateral ACAs are interconnected with the anterior communicating artery (AComA), while the posterior communicating arteries (PComA) interconnect the ICA with the ipsilateral PCA [2].

Deviations from this typical configuration have been widely reported in the current literature. Although isolated variants can be frequent, coexistence of several variations of the cerebral arterial circle are rarely reported in the current literature [1, 6, 8].

Herein, we are adding another complex and unique configuration of the cerebral arterial circle to the current literature.

## Materials and methods

During examination for possible cerebrovascular pathology, the scan of an 87-year-old female patient was further investigated. A magnetic resonance angiography (MRA) was examined at three Tesla (3T), while the imaging protocol was performed using the time-of-flight technique (TOF) with a Philips 3T Achieva TX MRI scanner (Philips, Best, the Netherlands) with an 8-channel head coil. The scans were documented using Horos software version 3.3.6 (Horos Project). Findings were derived from the multiplanar reconstruction of the axial, coronal, and sagittal slices and their three-dimensional volume reconstruction.

### Anatomical findings

**Fig. 1:**
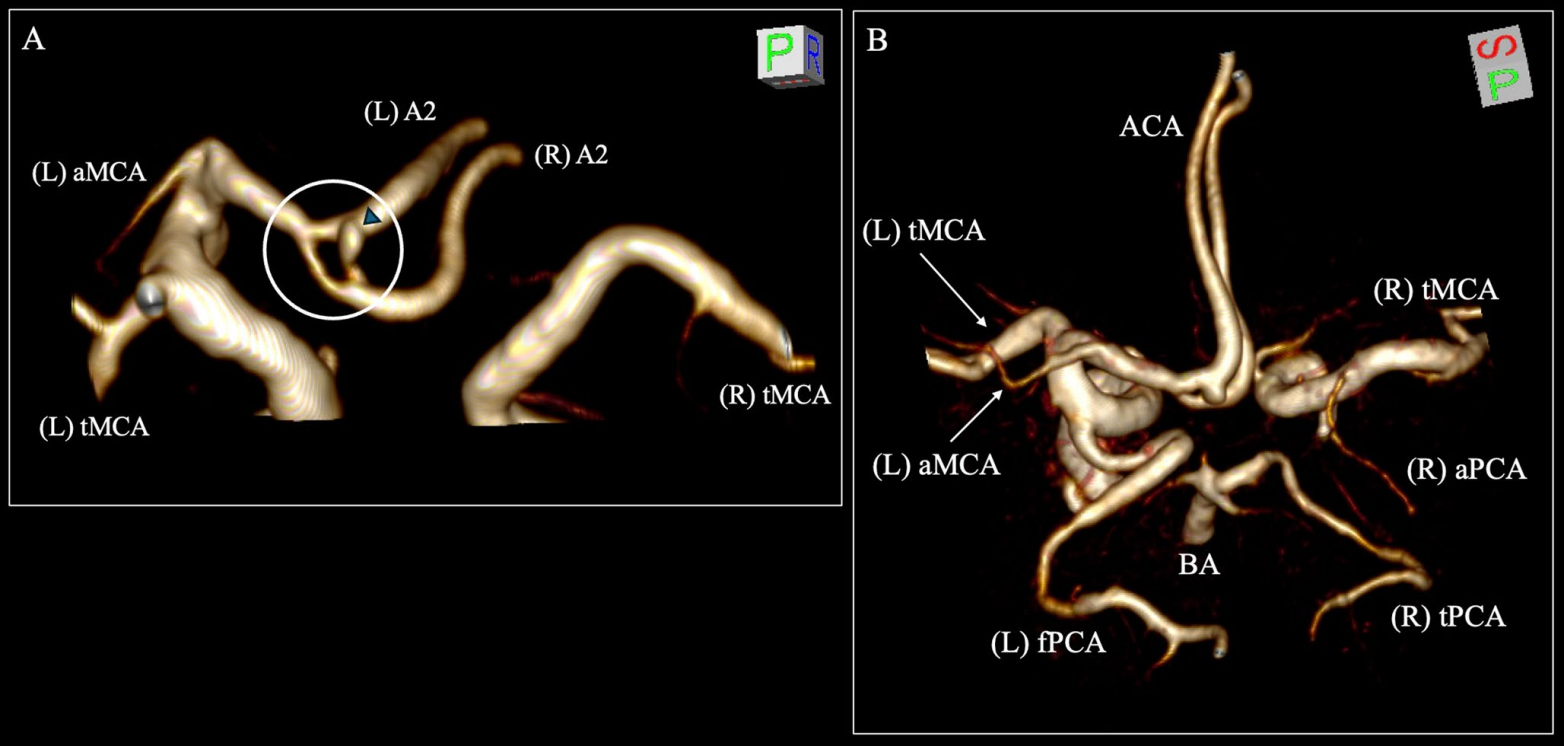
Three-dimensional reconstruction of the cerebral arterial circle of the patient. **A** The arterial ring (white circle) at the origin (double origin) of the A2 segment of the left anterior cerebral artery (ACA). There is also an infundibular dilation at the arterial ring (blue arrow). **B** The left accessory middle cerebral artery (aMCA) and the right accessory posterior cerebral artery (aPCA) are observed. tMCA- typical middle cerebral artery, fPCA- fetal posterior cerebral artery, tPCA- typical posterior cerebral artery, BA- basilar artery, ACA- anterior cerebral artery, L- left, R- right

Within the anterior circulation, the left ICA typically gave off the left MCA and the left ACA. At the junction between the left ACA and the AComA, an arterial ring (diameter: 2.59 mm) was identified at the origin of the A2 segment, indicative of an A2 double origin (Fig. 1A). Moreover, an accessory MCA (diameter: 1.71 mm) was observed originating from the proximal left A1 segment (Fig. 1B). However, contralaterally, the right ICA did not provide the right ACA; thus, it was considered A1 aplasia. The right A2 segment was supplied from the left ACA (Fig. 1A).

On the posterior circulation, the left PCA originated from the ipsilateral ICA, indicative of a fetal-type PCA. The left P1 segment was hypoplastic (Fig. 1B). The right PCA was typically originated from the BA, while no PComA was observed connecting the PCA with the ICA. A duplicated PCA (diameter: 1.55 mm) originated from the right ICA, supplying the temporal branch (Fig. 1B).

## Discussion

The most interesting finding of this report is the ACA-AComA complex arterial ring in coexistence with the accessory MCA and PCA duplication that creates a unique configuration.

The embryological development of the ACA-AComA complex provides a critical framework for understanding these unique configurations. During the early prenatal period, specifically in embryos of 11.5–18 mm, a plexiform anastomosis exists as a precursor to the mature AComA [4, 10]. Typically, this network undergoes regression to form a single vessel. However, an incomplete fusion or partial persistence of this embryonic plexiform anastomosis results in the wide range of variations observed in the AComA, making it the most variable segment of the cerebral arterial circle [4, 10]. In our case, the unique arterial ring at the junction of the left ACA and AComA likely represents a partial persistence of this embryonic plexus.

ACA-AComA complex variations are frequent. In our previous meta-analyses, we identified the AComA variations with a pooled prevalence of approximately 33% [3], while the ACA variants with a pooled prevalence of 6.25% [4]. Neither of the two systematic reviews depicted such an arterial ring indicative of A2 segment double origin. Recently, Uchino and Andoh [5] reported the first case of such variant. They observed arterial rings at the origin of A2 segments of bilateral ACAs with the absence of unilateral A1 segment. Thus, our case is the second representative of this variation; however, Uchino and Andoh [5] case did not have other cerebral variants.

MCA morphological variants are infrequent. Uchino et al. [7] performed one of the first comprehensive studies, with a prevalence of 3.8%. MCA variations can be fenestration, double origin, duplication or accessory forms. It is especially important to differentiate between duplicate and accessory forms. A duplicate MCA (prevalence: 2.1%) is a supernumerary vessel originating from the ipsilateral ICA, while an accessory MCA (prevalence: 1.2%) is a supernumerary vessel originating from the ipsilateral ACA [7]. Therefore, the current case corresponded to an accessory MCA.

PCA morphological variants are also infrequent. Uchino et al. [9] classified them into fenestration, accessory, complete duplication and replaced PCA. These three variants should be carefully diagnosed. The accessory PCA (prevalence: 0.46%) corresponds to a vessel originating from the ipsilateral ICA, indicative of a hyperplastic anterior choroidal artery, and supplying part of the PCA territory. In this case, the PComA is typically identified. The PCA complete duplication (prevalence: 0.04%) corresponds to a similar variation; however, there is no connection with the typical PCA with the PComA being absent. The replaced PCA (prevalence: 0.09%) corresponds to a vessel originating from the ipsilateral ICA, indicative of a hyperplastic anterior choroidal artery, and supplying the whole PCA territory [9]. Therefore, the current case corresponded to a duplicated PCA.

It is also important to refer to similar cases of the current literature with combined cerebral variants. Rusu et al. [1] reported a case of coexisted duplicate PCA, fenestrated BA and ACA. Moreover, Uchino and Irie [6] identified a case of combined accessory MCA and PCA, similar to the current case. Lastly, in the current case, we identified double origin of the A2 segment (ACA-AComA arterial ring), accessory MCA and PCA.

The clinical significance of these combined variations lies in the altered hemodynamics and the potential risks they pose during neurosurgical or endovascular procedures. First, the presence of unilateral A1 aplasia compromises the integrity of the circle, rendering the AComA complex the primary pathway for collateral circulation; this configuration increases hemodynamic stress and is associated with a higher prevalence of aneurysm formation [3]. Second, the arterial ring at the A2 origin, while rare, creates a diagnostic pitfall as it may mimic an aneurysm on radiological imaging or predispose the site to aneurysm formation itself [5]. Third, the accessory MCA, although serving as a potential collateral blood supply to the frontal lobe in cases of MCA occlusion, is clinically relevant because aneurysms can develop at its origin [7]. Finally, the identification of the accessory PCA is surgically critical; this vessel, representing a hyperplastic anterior choroidal artery, supplies the temporal branch of the PCA. Misidentifying this variant as a standard anterior choroidal artery or PComA could lead to inadvertent sacrifice during temporal lobe or sellar approaches, resulting in ischemia within the PCA territory [9]. Consequently, careful observation of MRA volume-rendering images is essential to identify such complicated arterial variations and prevent intraoperative complications.

## Conclusion

We reported a unique and complex configuration of the cerebral arterial circle characterized by the coexistence of an arterial ring at the ACA-AComA junction, an accessory MCA, and a PCA duplication. Although exceedingly rare, such anatomical possibilities enhance the importance of careful preoperative assessment with the use of angiographies.

## Supplementary Information

Below is the link to the electronic supplementary material.

Supplementary Material 1: Three-dimensional reconstruction of the cerebral arterial circle of the patient.

Supplementary Material 2: Three-dimensional reconstruction of the cerebral arterial circle of the patient focusing on the arterial ring of the anterior cerebral artery.

## Data Availability

Please contact the authors for data requests.
